# Molecular basis of Leigh syndrome: a current look

**DOI:** 10.1186/s13023-020-1297-9

**Published:** 2020-01-29

**Authors:** Manuela Schubert Baldo, Laura Vilarinho

**Affiliations:** Newborn screening, metabolism and genetics unit - human genetics department, Instituto Nacional de Saúde Doutor Ricardo Jorge (INSA), Porto, Portugal

**Keywords:** Leigh syndrome, Leigh-like syndrome, NARP, MILS, OXPHOS, Review

## Abstract

Leigh Syndrome (OMIM 256000) is a heterogeneous neurologic disorder due to damage in mitochondrial energy production that usually starts in early childhood. The first description given by Leigh pointed out neurological symptoms in children under 2 years and premature death. Following cases brought some hypothesis to explain the cause due to similarity to other neurological diseases and led to further investigation for metabolic diseases. Biochemical evaluation and specific metabolic profile suggested impairment in energy production (OXPHOS) in mitochondria. As direct approach to involved tissues is not always possible or safe, molecular analysis is a great cost-effective option and, besides biochemical results, is required to confirm the underlying cause of this syndrome face to clinical suspicion. The Next Generation Sequencing (NGS) advance represented a breakthrough in molecular biology allowing simultaneous gene analysis giving short-time results and increasing the variants underlying this syndrome, counting over 75 monogenic causes related so far. NGS provided confirmation of emerging cases and brought up diagnosis in atypical presentations as late-onset cases, which turned Leigh into a heterogeneous syndrome with variable outcomes. This review highlights clinical presentation in both classic and atypical phenotypes, the investigation pathway throughout confirmation emphasizing the underlying genetic heterogeneity and increasing number of genes assigned to this syndrome as well as available treatment.

## Introduction

**Fig. 1:**
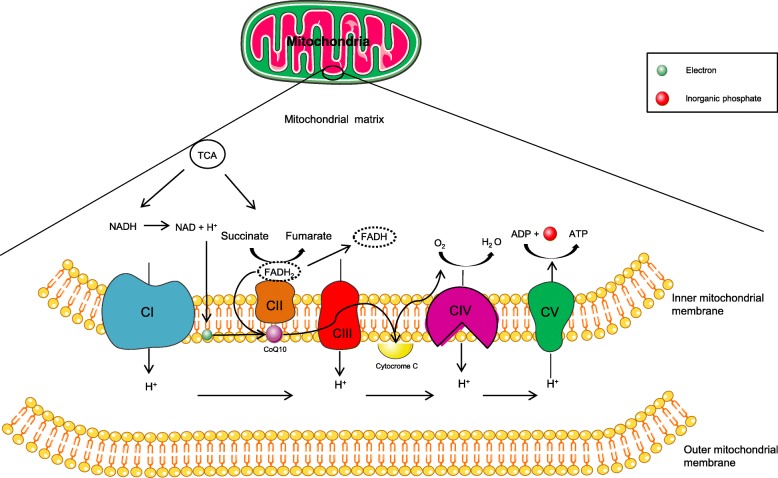
Representation of OXPHOS system in mitochondria. Energetic metabolism produces intermediates that can be used by cytochromes and be submitted to oxidation-reduction states producing electrons and in last step promoting ADP association to an inorganic phosphate resulting in ATP. Elaborated with Servier Medical Art

Mitochondria are a very particular multitask organelle with their own functioning. Composed by two membranes and a circular DNA (mtDNA), they command cellular energy production [1]. The metabolism and biocycle obey a self-signaling instead exclusive nuclear control. Mitochondria are capable of dividing themselves and producing their own material, including mtDNA products, to support their and major cell issues, requiring a refined and highly specialized arsenal to achieve optimal balance between replication and proteomic machinery in DNA synthesis [2]. They are responsible for energy production through tricarboxylic acid cycle (TCA) and electron transport chain (ETC), that is the main feature to generate ATP in the aerobic metabolism through an oxidative phosphorylation (OXPHOS), depending mainly on their inner membrane integrity to accomplish the entire process [3] (Fig. 1). ETC occurs with electron transport in changing membrane potentials, ion flux associated with cofactors, as riboflavin and coenzyme Q10, and generate ATP to sustain cellular demands. Some organs, like muscles and brain, require more energy to maintain their homeostatic functioning. Any damage in OXPHOS will diminish considerably energy supplies in high energy demand organs and it might produce signs and symptoms. Replication and homeostasis are a dynamic bioprocess and may lead to accumulation of pathogenic variants. These organelles have a peculiar condition to display heterogeneous presentation of DNA. There might be normal (wild-type) mtDNA along with mutated mtDNA with different rates between organs, even in the same mitochondria, in the same organism: this condition is defined as heteroplasmy [4]. Mitochondrial genome is inherited down maternal line, meaning that the offspring is identical in mtDNA. Although natural precautions are provided, such as bottleneck phenomenon, mtDNA is vulnerable to accumulation of novel mutations and it might enhance along time [5, 6]. When counting these quantities they may vary between descendents and organ to organ in the same organism, producing different percentage of energy and possibly clinical expression. Regarding their flaws they are classified in point mutations or defects of maintenance which may be depletion or multiple DNA deletion types, linked to variants that interfere quantitatively or qualitatively in mtDNA synthesis. The DNA maintenance defects are linked to nuclear DNA (nDNA) variants inherited in autosomal dominant or recessive manner [7]. In this context, there are authors that defend their similarity in physiopathology discouraging group separation but one *continuum* of the same [8]. Therefore, mitochondrial diseases may be due to any interference in ATP metabolism somehow affecting the OXPHOS process.

## Clinical presentation

**Fig. 2:**
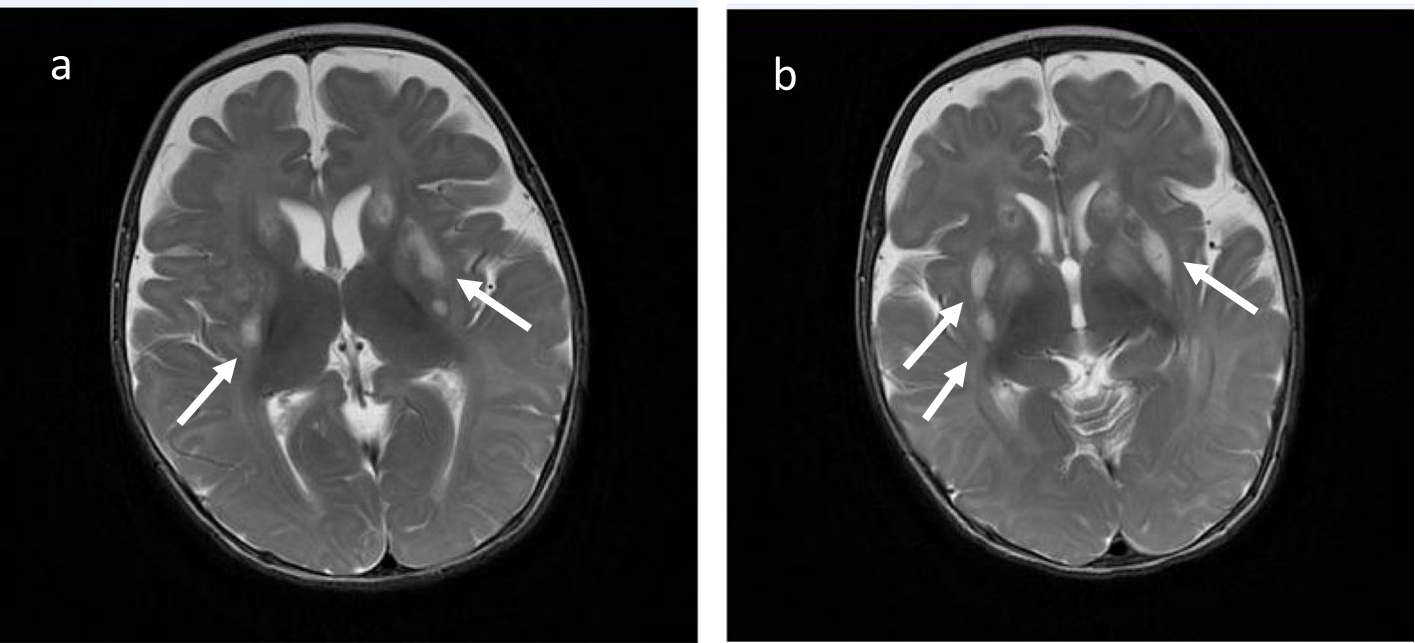
Brain MRI images in axial T2-weighted acquisition (**a**, **b**) demonstrating abnormal sign in bilateral basal ganglia (white arrows), which is a common finding of Leigh Syndrome. Case courtesy of Dr. M. Venkatesh, Radiopaedia.org, rID: 27512

Mitochondrial disorders of energy generation are the most frequent group of inborn errors of metabolism due to a dysfunction in OXPHOS (Fig. 2). They comprise distinct types of transmission (X-linked, autosomal, mitochondrial/maternal) due to nuclear, mitochondrial genome or in the interplay between two genomes resulting in great heterogeneity and wide range of presentation [9], from prenatal to adulthood. In childhood the most common presentation is Leigh syndrome (LS) or subacute necrotizing encephalopathy. It was first described by Denis Archibald Leigh in 1951 through *postmortem* investigation of a 7-month-old boy with progressive neurologic symptoms [10, 11]. Since first description based on histopathological findings, many tests tried to support the diagnosis revealing a damage in mitochondrial metabolism [12]. Currently, over 75 nuclear genes are related to this syndrome and still in expanding scenery [13]. Its variable onset brought up the diagnosis to be present in more age groups. The classical form usually starts before 2 years of age, even manifesting in neonatal period, and presents with hypotonia, epilepsy, respiratory stress, neurodevelopmental delay, ataxia and lactic acidosis. Typical neuroimaging reveals symmetrical hyperintensity in T2-weighted images in magnetic resonance imaging (MRI) in basal ganglia and/or brainstem with a lactate peak in affected areas in spectroscopy (Fig. 2). In addition cerebral white matter, thalamus, spinal cord and *cerebellum* may be affected as well [14]. Such identifiable imaging findings are a response to OXPHOS dysfunction and lower ATP generation. The neurons of higher energy demand in dysfunctional stock of ATP trigger a stress cascade culminating in gliosis and vacuolization of neuronal tissue over time [15]. The late-form presents in more heterogeneous way, with behavioural/psychiatric findings, intellectual decline, movement disorders, headaches, memory loss or even mimicking a multiple sclerosis-phenotype [16]. Although highly specific to central nervous system there may be non-neurological symptoms. This condition is classified as Leigh-like syndrome (LLS) and comprises cardiac, hepatologic, gastrointestinal, hematological abnormalities and dysmorphic features [17]. As the main basis is impairment in mitochondrial ATP production, stress situations (infection, prolonged fasting, vaccine, anesthesia, surgery) may trigger an acute decompensation due to increased energy demand and produce symptoms. These patients are vulnerable and must undergo to careful investigation. Neuroimaging can provide valuable information and help medical group making decisions, except it requires in certain cases the patient to go through an anesthetic procedure and it might be deleterious even if successfully accomplished. Anesthetic components, as well as some antiepileptic drugs, may reduce even more the metabolism failing in producing ATP [18]. Relatively to imaging studies and considering the genetic heterogeneity involved, this pathology may display different findings. Complex I deficiency usually presents basal ganglia involvement, stroke-like lesions (mtDNA) and leucoencephalopathy (nDNA). Complex IV – cytochrome *c* cxidase – deficiency resembles complex I imaging finding*s*. Complex V deficiency, mainly ATP6 and ATP8 related genes, exhibits basal ganglia and brainstem involvement with cerebellar atrophy but no stroke-like lesions, which may shorten the investigation. The translation defects might exhibit other findings such as: calcification in basal ganglia, cortical and cerebellar atrophy and stroke-like lesions in Mitochondrial Myopathy, Encephalopathy, Lactic Acidosis and Stroke-like episodes (MELAS). Pontocerebellar hypoplasia is seen in *RARS2* and pyramidal tract and spinal cord involvement associated with a lactate peak in spectroscopy are well associated to *DARS2*. A specific and occasionally reversible white matter dystrophy sparing the periventricular rim is suggestive of *EARS2* involvement and severe cortical atrophy with putaminal hyperintensity is relevant for *FARS2* involvement [19–21].. The maintenance defects display cortical and cerebellar atrophy with stroke-like lesions in *POLG* association [22], as *SUCLA2/SUCLG1* display basal ganglia involvement and cerebral atrophy [23]. In case of having such suggestive characteristics, molecular diagnosis may be easier. As far as the overall investigation it includes clinical records, family history, physical examination, biochemical evaluation in body fluid samples (lactate, pyruvate, glucose, blood gas profile, amino acids profile in blood/ spinal fluid and organic acids in urine), specific muscular tissue analysis (assessment of mitochondrial respiratory chain complexes) and ideally genetic identification of the responsible variant. As biochemical approach is not always elucidative or safe, genetic evaluation has become an eligible initial step in investigation especially if a pattern of signs and/or symptoms is found suggesting a specific condition or complex/complexes deficiency. Despite ongoing trials and progress, there is no curative treatment available, only towards supporting symptoms and avoiding metabolic crisis.

## OXPHOS defects

Complex I (ubiquinone oxireductase - NADH): the first mitochondrial complex is known to be the most associated to mutations in mitochondrial diseases, with several LS related genes described so far, being the largest mitochondrial complex involved in ETC [24]. The LS spectrum of presentation is wide regarding the underlying variant, presenting with ataxia, oculomotor apraxia, seizures, neurodevelopmental delay, dystonia, failure to thrive, cardiomyopathy and apnea. Leukoencephalopathy is basically a signature feature of complex I deficiency [11]. There are some mainly related genes to complex I assembly factors and subunits with LS phenotype previously described, new related-genes with variable phenotypes and new mutations in previously related genes, confirmed through assertive functional methods (Tables 1 and 2).

**Table 1:** Nuclear genes involved in OXPHOS causing Leigh and Leigh-like syndrome

Biochemical defect	Associated nuclear genes
Complex I Deficiency	*NDUFA1, NDUFA2, NDUFA9, NDUFA10, NDUFA12, NDUFS1, NDUFS2, NDUFS3, NDUFB8, NDUFS4, NDUFS7, NDUFS8, NDUFV1, NDUFV2*
Complex II Deficiency	*SDHA*
Complex III *Deficiency*	*UQCRQ*
Complex IV Deficiency	*NDUFA4, COX8A*
Assembly factors deficiency	
Complex I	*NDUFAF2, NDUFAF4, NDUFAF5, NDUFAF6, C17ORF89, FOXRED1, NUBPL*
Complex II	*SDHAF1*
Complex III	*BCS1L, TTC19*
Complex IV	*SURF1, COX10, COX15, SCO2, PET100*
Cofactors and biosynthesis	
Pyruvate Dehydrogenase Complex	*PDHA1, PDHX, PDHB, DLAT, DLD*
Biotinidase Deficiency	*BTD*
Thiamine Deficiency	*TPK1, SLC19A3*
Lipoic acid	*LIPT1, LIAS, BOLA3*
Amino acid	*HIBCH, ECHS1*
Coenzyme Q10 Deficiency	*PDSS2, COQ9*

**Table 2:** Nuclear genes involved in other mitochondrial functions causing Leigh and Leigh-like syndrome

Mitochondrial function	Associated nuclear genes
Mitochondrial DNA maintenance	*FBXL4, POLG, POLG2, SUCLA2, SUCLG1, TWNK*, *SLC25A4, MPV17*
Mitochondrial translation	*GTPBP3, TRMU, EARS2, FARS2, IARS2, GFM1, GFM2, LRPPRC,TACO1, MTFMT, C12Orf65, MRPS34, MRPS39 (PTCD3), NARS2, TSFM*
Mitochondrial dynamics	*SLC25A46, DNM1L, MFN2, RRM2B*
Mitochondrial material import/export	*SLC25A19*
Membrane phosphocomponents	*SERAC1*
Sulfur dioxygenase	*ETHE1*
AAA oligomeric +/− ATPase	*CLPB, SPG7*
RNA import	*PNPT1*
RNA-specific adenosine deaminase	*ADAR, RNASEH1*
Nuclear translocation system	*RANBP2*
Nuclear pore complex	*NUP62*
Manganese transportation	*SLC39A8*

Baertling F. et al described a patient with *NDUFAF4* missense variants c.194 T > C (p.Leu65Pro) displaying an early-onset with neurodevelopmental regression*,* hypotonia, failure to thrive and irritability [25]. Biochemical profile showed hyperlactacidemia in plasma and cerebral spinal fluid (CSF). Brain imaging suggested LS, respiratory chain complexes analysis in fibroblasts demonstrated reduced complex I activity confirming WES results. Incecik F. et al confirmed a consanguineous late-onset LS presented with progressive ataxia and dysarthria with normal serum biochemical analysis but abnormal spectroscopy, in which a lactate peak was apparent in both *putamen* and right caudate topography [24]. *NDUFV1* homozygous variants c.1268C > T (p.Thr423Met) were identified pointing to a complex I deficiency. Previous descriptions for complex I include early-onset presentation displaying muscle hypotonia, spastic diplegia, oculomotor disfunction, ataxia, lethargy, without leukodystrophy in brain imaging. Lou X. et al presented an early-onset case in a 7-month-old boy with torticollis, brain MRI compatible with LS, who evolved to metabolic crisis and progressive lesions in basal ganglia in imaging studies [26]. Whole Exome Sequencing (WES) identified missense variants c.418 C > T (p.Arg140Try) – new variant - and c.595 C > T (p.Arg199Try) in *NDUFS3* confirmed by Sanger sequencing and tested in B-lymphocytes of the *trio*. Confirmed segregation pattern, both variants were characterized implicated in reducing complex I activity. Beyond nuclear control, there are also mtDNA variants that reduces complex I functioning. The codifying mtDNA genes as mitochondrial encoded ND genes *MTND* (1–6) can interfere in complex I electron pumping and therefore produce a compatible phenotype. *MTND1* m.3697G > A previously reported in MELAS is suggested to be implied as well [27]. Ma YY. et al screened Chinese patients with LS and identified previously reported variants as m.10191 T > G in *MTND3* causing the phenotype in their group and suggested to be the most frequent variant in their population [28]. Another variant reported by Ruiter EM. et al to be frequent is m.13513 G > A in *MTND5*. A biochemical proven complex I deficiency in association with optic atrophy and/or a cardiac conduction defect syndrome was found and described in their cohort [29]. Despite its occurence in MELAS, Leber’s Hereditary Optic Neuropathy (LHON) and overlap of these two, Sudo A. et al found 7% of this variant’s prevalence in their study [30]. The same situation occur in m.14487 T > C in *MTND6* broad phenotype possibilities, related to severe LS in homoplasmy rates and in lower rates to progressive myoclonic epilepsy in adulthood [31].

Complex II, III and Coenzyme Q10: far less common than complex I (around 8%). Complex II - SDH (succinate dehydrogenase) has four subunits, the most important codified by *SDHA* and *SDHB* genes, being *SDHA* the most implicated. Patients may present with early onset and classic rapid progression or late form involving Kearn-Sayres, dementia, myoclonic epilepsy and surviving to late childhood [32]. Complex III (ubiquinol:cytochrome c oxidoreductase) is rare to be implicated to mitochondrial diseases in general and three nuclear genes mainly involved in LS – *UQCRQ*, an assembly protein, *TTC19*, an assembly factor and *BCS1L* [33]. *BCS1L* is the most frequent out of three, a complex chaperone that encodes ATPases necessary for complex assembly. *BCS1L* deficiency often manifests as GRACILE-like/ LLS combining low weight at birth, renal and hepatic dysfunction, hypotonia and developmental delay. The previous described are encompassed in an intermediate dysfunction phenotype, also named “myopathy *BCS1L* phenotype” [34]. *UQCRQ* codifies a subunit of complex III, and is related to early-onset severe LLS identified in a consanguineous Israeli Beduin kindred family. Descriptions of *TTC19* range from psychiatric symptoms and ataxia [35] to global neurodevelopmental delay and language regression in early childhood [36]. In a report, it is described that the majority of patients with complex III deficiency related to *TTC19* have consistent neuroradiological findings to LS [14]. Coenzyme Q10, or ubiquinone, is a vital ETC component that promotes electron transport via flavoproteins from complex I and II to III [37]. This deficiency might manifest with ataxia, migraine, ptosis, deafness, lactic acidemia and display neuroradiological findings of LS. Signs recognition must be carefully taken under consideration when supplementing this cofactor. Van Maldergem L*.* et al compared two sisters with confirmed deficiency of Coenzyme Q10 (CoQ10) in muscle tissue, with important recovery of abilities, behaviour and growth parameters with 300 mg/day [38]. Glover EI. et al tried a randomized trial with CoQ10 to mitochondrial disorders but no meaningful results were observed especially in higher doses, leaving questions around supplementation dose when no proven deficiency [39]. The ideal is testing CoQ10 in muscle tissue directly measuring ETC functioning and quantifying the cofactor or through molecular diagnosis related to this cofactor [37].

Complex IV (Cytocrome *c* oxydase - COX): The *SURF1* variants are the most common playing part in Complex IV assembly, counting majority of cases described so far. Despite relatively homogeneous clinical findings like hypertrichosis even in presymptomatic state [40], neuroimaging may display uncommon features such as leukodystrophy and atrophy [14]. They appear to have better prognosis than other syndromes caused by genes implied in complex IV metabolism, such as *ETHE1* and *LRPPRC*. The *ETHE1* is related to ethylmalonic encephalopathy (OMIM #602473) and may manifest as LS due to accumulation of sulfide, which reduces complex IV functioning and justifies the resemblance. *LRPPRC* variants are related to first description in French-Canadian population and present a characteristic phenotype, including facial dysmorphic features, liver disease and recurrent lactic acidosis [41]. In addition to interfering in cytochrome *c* oxidase function, *LRPPRC* interposes in global mitochondrial translation and mitochondrial RNA (mRNA) maturation, in ATP synthase complex and lipid metabolism in peroxisomes, playing more roles but presenting mainly as complex IV deficiency. Such findings may highlight the phenotype difference from other LS causes [42]. Previously related to complex I, *NDUFA4* is recently proven through functional studies to codify a complex IV subunit [43], presents as late-onset form with learning disabilities, epilepsy and survival into adulthood [14].

Complex V (ATP synthase): Unlike other complexes, complex V is mainly under mtDNA control and represents the last OXPHOS step – generating ATP. The two subunits (F0 and F1) produce a conformational change when receiving ADP, coupling to inorganic phosphate (Pi) and converting ADP into ATP. Some variants are well related to mitochondrial diseases and even LS. The *MTATP6* m.8993 T > G/C variant, that damages the F0 portion of ATPase [44], is associated to Maternally Inherited Leigh Syndrome (MILS) and to Neuropathy, Ataxia and Retinititis Pigmentosa (NARP) syndrome with basically the heteroplasmy rates defining the phenotype. Considering rates over 60% and lower than 85% it is expected a NARP syndrome phenotype; 85% or more it is expected a MILS phenotype, although variations in these values and phenotypes have been reported. Besides heteroplasmy rates a common finding related to this variant is hypocitrullinemia in amino acids evaluation, which may shorten the investigation as Vilarinho L. et al demonstrated in a report that two out of three families had low levels of citrulline [45] and Lopes T. et al reported in a patient [46]. In addition to *MTATP6* are m.9176 T > G and m.9185 T > C, described to be associated with late-onset cases [47]. In a Portuguese study of m.9176 T > C, Carrozzo R. et al investigated two sisters with LS where one of them had low levels of this metabolite, displaying a new possibility to LS with hypocitrullinemia as well [48].

**Fig. 3:**
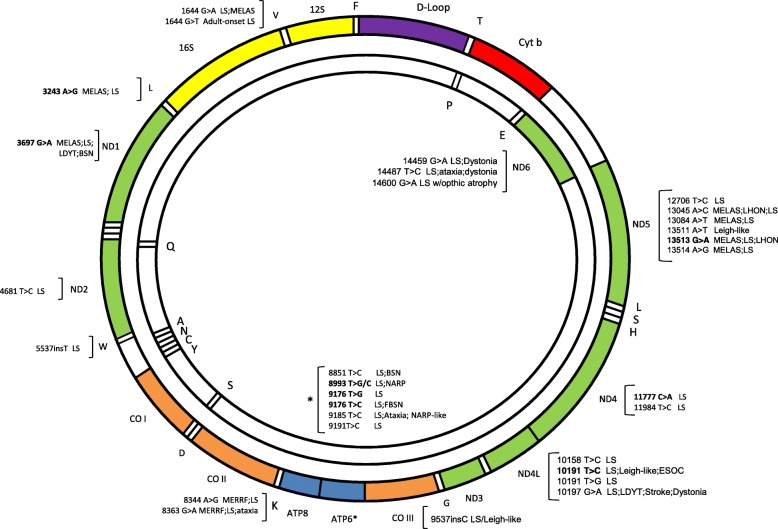
Mutations in mitochondrial genes causing Leigh and Leigh-like syndrome are represented and described in mitochondrial genes. The most frequent mutations related to Leigh and Leigh-like appear in bold

Combined deficiencies: Since mtDNA encodes components of complexes I, III, IV and V, a pathogenic variant that affects mtDNA maintenance can result in combined OXPHOS impairment. Whereas deletion syndromes are related to rearrangements in DNA, depletion syndromes produce reduction in DNA copy numbers [49]. New reports include LS to the nomenclature due to overlapping phenotypes as genes associated with mtDNA deletion or depletion syndromes were identified in these patients [50]. The most frequent causes of mtDNA depletions are related to *SUCLA2* and *SUCLG1*. By affecting succinyl-CoA synthetase, an important step of TCA, they manifest mild to elevated levels in methylmalonic acid in organic acids profile and reports suggest that this metabolite is good in surveillance and investigating the disease [14]. *SUCLA2* tends to present as early-onset LLS syndrome with muscular atrophy and sensorineural hearing impairment, while *SUCLG1* can express in a severe and fatal phenotype including harsh hyperlactacidemia and pronounced liver disfunction. However, descriptions of indistinguishable phenotype between them have reported, adding a confusing factor to investigation [51]. *SERAC1* is responsible for cholesterol trafficking and remodeling phosphatidylglycerol 34:1 to 36:1, a structural cell membrane component cardiolipin. *SERAC1* is related to MEGDEL syndrome, a progressive syndrome defined by 3-Methylglutaconic aciduria, Deafness, Encephalopathy and LLS [52, 53]. Other important function in mitochondria is DNA translation, resulting in mitochondrial nucleic acids production. It is performed by modifying proteins (mt-tRNA) and constitutes another group implied in combined deficiencies. *MTFMT* was the first mt-tRNA reported to be responsible for LS, being c.626C > T a frequently identified variant in this group [14]. Subsequently, came over others like *MTTL1* and *MTTK* to be linked to LS: m.3243A > G *MTTL1* responsible for MELAS and m.8344A > G *MTTK* related to Myoclonic Epilepsy with Ragged Red Fibers (MERRF). After production of nucleic acids, mtDNA must undergo through proteins synthesis to be polymerized, an event under *POLG* control [54]. Despite directly involved in mitochondrial bioprocesses, *POLG* is not maternally inherited but in an autosomal dominant or recessive manner. *POLG* is related to Alpers-Huttenlocher Syndrome (severe encephalopathy, seizures, and liver failure) but accordingly to the variant might produce various phenotypes, like Alpers, Progressive External Ophthalmoplegia (PEO) and LS. Its deficiency produces a LLS phenotype, which includes hepatic dysfunction as Alpers [8] and may include hypertrophic olivary degeneration as in *SURF1* deficiency [55]. As *POLG*, other rare genes related to mtDNA maintenance but non-related to LS are currently proven to express the phenotype. Souza, PVS. et al published in a recent study in a heterogeneous age group (0-69y) uncommon responsible genes and new variants in formerly related to LS/LLS genes: *SPG7*, a mitochondrial protease, was related to basal ganglia abnormalities never referenced before; *RRM2B,* a nucleic acid supplier to mitochondrial machinery, evidenced parkinsonism and LS features in imaging in an unusual phenotype. *MFN2, a* protein related to mitochondrial dynamic bioprocesses, displayed basal ganglia lesions with movement disorders and deafness. *TWNK,* a helicase involved in replicating mitochondrial genome, presented with stroke-like episodes and never before confirmed LS phenotype. *MPV17,* an essential protein to keep nucleic acids pool, is related to Navajo neurohepathopathy not comprising LS features previously. *SLC25A4,* an inner transmembrane mitochondrial channel to ADP-ATP, displayed unusual central nervous system abnormalities consistent with LS. *RNASEH1*, an endonuclease to degrade RNA, manifested late-onset LS with progressive parkinsonism, *pes cavus* and cerebellar ataxia [56]. Even though the most common causes in mtDNA LS/LLS are point mutations or small deletions [7], DNA rearrangements (deletion) and depletion syndromes are rising in number accounts to mitochondrial disorders due to new genetic tools. Hence, latest reports phrase that NGS have enabled diagnosis and expanded the previously known LS/LLS phenotypes (Fig. 3).

## Other defects non-OXPHOS

Biotine-responsive basal ganglia disease (BBGD)/ *SLC19A3* deficiency: thiamine pyrophosphate (TPP) is an important cofactor of more steps in energy production playing main role in several pathways in neurometabolism and SLC19A3 is a specific biotin-dependent thiamine transporter in basal ganglia system [57–59]. The lack of thiamine might produce three phenotypes along life: early-onset LS, the BBGD in childhood and Wernicke Encephalopathy in adulthood [60]. BBGD mainly presents with hyperlactacidemia, vomiting, seizures and encephalopathy. The CNS free-thiamine dosage is frequently in low quantities nearly to absence, which can enhance diagnosis suspicion [61]. The neuroimaging is consistent with LS except cortical and subcortical abnormalities that are unusual for classic LS presentation. In *SLC19A3* deficiency thiamine and biotin supplementation might improve symptoms and have promising outcomes [57].

Pyruvate Dehydrogenase Complex deficiency (PDHc): pyruvate dehydrogenase complex that is composed by units E1, E2 and E3 - the E1-alpha and E1-beta require active thiamine (TPP) which interferes in converting pyruvate in acetyl-CoA in TCA, the main substrate to ETC. In a deficiency state, the exceeding pyruvate is transformed in lactate forbidding the ETC to move forward with glycolysis [62, 63]. The X-linked PDHc deficiency (PDHA1) is related to *PDHA1* and it is the most frequent PDHc deficiency. Variants in *PDHA1* produce high lactate, pyruvate and alanine levels, failure to thrive, seizures. Basal ganglia and *corpus calosum* abnormalities are common feature. Treatment is based in dietary modification by enhancing calories through lipids, thiamine supplementation in high quantities and controlling lactate/pyruvate levels.

Lipoyltransferase 1 deficiency (*LIPT1*): lipoic acid is necessary to many assembly complexes and intermediate mitochondrial metabolism in ETC to generate ATP [64]. The metabolism to its active form requires active thiamine as a cofactor, implying integrity in related pathways to succeed and its deficiency interferes in other pathways like glycine clivage system, PDHc, α-ketoglutarate dehydrogenase (KGDH) and branched-chain α-ketoacid dehydrogenase complex BCKDH - E3 form. Its deficiency produces metabolic acidosis due to hyperlactacidemia, normal/elevated branched-chain amino acids and glycine levels in serum and/or CSF. Reposition of lipoic acid is not effective, restricting specific treatment options [65].

## Biochemical investigation

As first approach to a suspicion of LS it is reasonable to evaluate the possible affected pathways, considering energy supplies and metabolic intermediates generated in response. As so, blood gas analysis, lactate and pyruvate, glucose and electrolyte profile are the main metabolic basis. LS include normal/raised lactate/pyruvate levels in plasma and/or cerebral spinal fluid (CSF) and may present with metabolic acidosis and hypoglycemia in decompensation status. Specific profiles as amino acids and organic acids are helpful and may give hints of the underlying cause. Amino acids profile in plasma may show alanine or citrulline alterations, compatible as well to the syndrome. Alanine is produced in response to high lactate levels and citrulline is diminished in a specific mtDNA variant – m.8993 T > G, shortening investigation [66, 67]. A specific metabolite search in plasma, such as thiamine pyrophosphate (TPP), could allow a thiamine transporter deficiency diagnosis due to *TPK1*, a treatable cause of LS [68]. Organic acids profile may reveal specific pattern of acids in urine suggesting the blocked pathway (methylmalonic, 3-methylglutaconic) or even intermediates of TCA, often present in mitochondrial defects. In CSF evaluation a free-thiamine dosage would help the investigation of thiamine transporter deficiencies, such as *SLC19A3*, another treatable cause of LS related to thiamine transporter deficiency [61]. Lactate levels represent the local metabolism status being a promising finding [11]. As it relates sensitively to central nervous system (CNS) metabolism it is more specific than peripheral lactate levels that are under many confusing factors, such as sample collection manner, material stocking and others. Raised levels may also be identified in spectroscopy but it is not understood yet the minimum quantity to be present to be identified [69] even in a more accurate investigation with proton acquired imaging. However, in many asymptomatic or undiagnosed cases spectroscopy may show precocious local metabolism impairment and lead the investigation towards metabolic etiology [70]. Some findings in neuroimaging are found to be suggestive of mitochondrial disease as stroke-like lesions, *striatum* commitment and brainstem alterations associated with lactate peak in spectroscopy [71]. Furthermore, as not always neuroradiologic features or biochemical approach are positive, especially for mitochondrial disorders, new biomarkers are been studied in order to better help in a less invasive manner such as tissue biopsy. FGF-21 (fibroblast growth factor 21) and GDF-15 (growth and differentiation factor 15) are serum cytokines produced in liver and muscle tissue that were found to be raised in mitochondrial diseases, particularly in muscle manifesting mitochondrial diseases, when compared to previously used biomarkers (lactate, pyruvate, lactate to pyruvate ratio and creatine kinase). In order to correlate circulating levels to disease and prove specificity and sensitivity parameters, some authors published data comparing them with each other and each of them to the standardized serum biomarkers. It was proposed that FGF-21 correlates well with disease severity and has sufficiently specific and sensitive to mitochondrial myopathies but few years later it was verified that nonmitochondrial heterogeneous groups also displayed raised levels of this component. Shortly after GDF-15 was suggested to have more sensitivity and specificity rates than FGF-21, a result showed especially in children, and associating their measurements would be better than isolated [72]. Yatsuga A. et al even described better statistic profile of GDF-15 over FGF-21 for severity and mitochondrial basis except for non efficient discriminative profile in cardiac muscular involvement etiology [73]. Lehtonen JM. et al reported raised levels of FGF-21 in mitochondrial translation defects and mtDNA deletions and correlated positively with disease severity being a possible biomarker to follow up [74]. Morovat A. et al demonstrated that FGF-21 displays considerable overlapping values between mitochondrial and nonmitochondrial diseases, increased levels in other conditions yet stability in repeated measurements to follow up procedures and important positive correlation in ophthalmoplegia in adults even suggesting to dismiss muscle assessment to produce a definite diagnosis [75]. Given the disagreement, a Russian group recently published results comparing mitochondrial disease groups with nonmitochoindrial controls for each biomarker and had no significant results in their cohort studies pointing raised levels in metabolic nonmitochondrial group, though mitochondrial group had higher values, which require further investigation of these biomarkers [76].

A more invasive and harmful possibility for investigation and diagnosis is tissue biopsy (skin, muscle). Although quite criterious in acquiring samples due to invasive collection procedure, histopathology for muscle sample and OXPHOS analysis for both muscle and skin fibroblasts are still an alternative to a biochemical assessment and are profitable in providing a diagnosis. Muscle samples should be processed right away for entire OXPHOS assessment or if not must be frozen and then complexes must be evaluated individually [77]. Despite precautions with technique and procedure (local anesthesia), OXPHOS assessment in muscle biopsy is preferable under general anesthesia. However, regarding clinical phenotype may add little contribution depending on mainly affected organ. To perform muscle biopsy in parallel to genetic evaluation is a practice that is required in cases of great clinical instability or rapidly progressive symptoms, such as classical Leigh syndrome presentation, in isolated muscle involvement disease and in cases that muscle biopsy could provide diagnosis and change or start specific treatment. The negative genetic evaluation or only confirmation of already disease causing genetic findings does not require simultaneously or prompt muscle evaluation [78].. Skin fibroblasts are an alternative to muscle in small children, less invasive, but in a heteroplasmy possibility they may result normal or inconclusive as well [11].

## Genetic evaluation

When it comes to DNA analysis there are alternatives to address the respective content. Mitochondrial DNA is a circular DNA, double-stranded, easier to access and sequence than nuclear DNA. The investigation decision relies in having a specific suspicion or not. Whenever identified a novel variant there has to be a prediction of its behavior in vivo. The bioinformatic tools are helpful in estimating these results in silico predictors as MutationTaster, PolyPhen-2, SIFT by performing an analysis and, therefore, providing an estimate result [79–81]. In children with early presentation the investigation in molecular matters should start in mitochondrial DNA. The most frequent mutation related to this phenotype in early childhood is m.8993 T > G/C related to maternally-inherited Leigh syndrome (MILS) and NARP syndrome [82]. The more time passes by, the more mitochondria are vulnerable to nDNA command, which may point first molecular investigation in late-onset groups to nDNA. In deletion/depletion syndromes, this rule is not valid. They depend on accumulation of abnormal variants in mtDNA, enhanced by time, to produce symptoms. It is known that the bottleneck phenomenon narrows the mutation loads by producing lower mitochondrial replication whenever higher mutant loads are present and the opposite as well, but it may be influenced at every cycle [2]. The particular relevance of onset is due to literature results demonstrating that in many suspicious or unclear cases the first genetic approach is NGS – gene panels, clinical exome sequencing, whole exome/genome sequencing. The difference is the coverage of genes in an individual analysis: 5000 genes in clinical exome and 20,000 in whole exome but still representing 2% of the DNA – the exomic (codifying) regions, where genome includes all codifying and non-codifying sequences [83]. In nDNA defects, it’s valuable to search or recognize signals and symptoms that strengthen possibilities, leading to one probable disease-causing gene. In case of specific phenotypic findings the search might be direct, shortening time for diagnosis [11]. When the phenotype is not well defined, a wide investigation is required. Nogueira C. et al published a report in use of NGS techniques for mitochondrial diseases in adults and children, displaying favorable results in gene-panel NGS assessment to provide molecular diagnosis to this selected group and suggesting the complementary investigation through exome in order to expand candidate genes to evaluation giving the panel limitation design and gene targets [84]. A recent report of a Brazilian group with variable age presenting diverse neurological features was assessed by magnetic resonance of the brain and spinal cord, biochemical profile, muscle biopsy and further genetic characterization by WES. Surprisingly the results came suggestive of LS/LLS by mtDNA-maintenance defects. The variants were not previously related to them, something a gene panel would have missed. A Polish group investigated pediatric patients that were selected to WES evaluation in a Health Center with mitochondrial diseases suspicion based on the Mitochondrial Disease Criteria. They reported 90% of positive results in their cohort especially in neonatal patients, a highly unstable age range and susceptible to severe and fatal clinical presentations [85]. In contrast, Taylor RW. et al analyzed 53 patients of two centers from distinct countries who had respiratory chain assessment in muscle biopsy with evidence of multiple respiratory chain complex deficiency but no pathogenic variant identified in mtDNA. WES was performed and 32 patients had results involving 18 candidate genes, including recurrent pathogenic variants and novel variants in mitochondrial genes that could possibly be disease-causing. The group described the negative identification of candidate variants and the difficulties of validating results of non-reported variants in literature until then leaving 21 patients undiagnosed [86]. In pediatric or rapidly progressive ill patients a correct diagnosis in fast performance is still challenging, even more in hospitalized ones. The need has stimulated new modalities of genetic evaluation such as the rapid Whole-Exome/Genome Sequecing (rWES/rWGS). Saunders CJ. et al explained the use of rWGS in Neonatal Intensive Care Unit patients using a rapid genome analysis promising less than 3 days to results. Crossing clinical information and candidate genes identified in rWGS the diagnosis was directed and time-shortened [87]. This technique might be promising as in hospitalized children demonstrating enhancing diagnosis, readjustment in treatment manners, saving costs of unnecessary hospitalization as Farnaes L. et al described, and even in approach to fetal hydrops in a Niemann Pick type C fetus [88, 89]. Although proven to be effective, rapid approaches are even rarer than the classic WES or WGS and might be questionable particularly in non mendelian inherited disorders identification [87]. The unsatisfactory or inconclusive outcomes like Taylor RW. et al described motivate emerging fields as the “omics” evaluation, namely transcriptomics, is one of them. The analysis of transcriptome, or the group of DNA transcripts, could explain the non mendelian inherited diseases and other modalities of expression (aberrant expression, aberrant splicing and mono-allelic expression) that exome and genome themselves would miss. Furthermore, WGS would detect more variants by analyzing the genome material but interpreting coding and non-coding new information enhance the challenge and require validation as well. Analyzing the RNA-sequencing (RNA-seq) in clinical practice would provide stronger evidence for WES and WGS results [83]. Despite robust findings in bioinformatic techniques the functional analysis must be taken in order to prove themselves or not, where more “omics” (ex: proteomics, metabolomics) tasks are welcome to take place and be a positive approach to effective diagnosis. Indeed, it requires a more accurate investigation to better comprehension and improve diagnosis effectiveness but still is an open door to be explored [90].

## Genetic counseling and prenatal diagnosis

LS has mitochondrial and nuclear DNA as possible causes of disease. Hence, it is important to define whether autosomal, X-linked or maternally inherited to genetic counseling and future decisions. If the mother has already lost a child to the disease or is known to harbour mtDNA mutation, it is well expected to undergo through testing. Prenatal diagnosis is feasible when there is possibly known causative mutations with well established physiopathology, segregation pattern and linkage studies [91, 92]. The objective is to provide an assessment of the risk to the fetus in developing disease antenatal or in childhood. When a mtDNA variant is involved there has to be a proper determination of heteroplasmy percentage in the mother by testing blood and urine sample and the fetus by chorionic villus biopsy testing (CVB). Even though assuming pathogenic rates above 60%, in many cases, it is not possible to assure that offspring will be affected considering possible changes in these numbers until birth [92, 93]. One variant that has stable mutation load levels throughout the gestation period and in most affected tissues including blood is m.8993 T > G/C, favoring proper diagnosis and counseling during pregnancy. Some articles reviewed different mtDNA variants, as the m.9176 T > C, and adopted more directive counseling in extreme mutation loads [94]. Considering that the mutant load may change during pregnancy and vary from tissue to tissue, careful advice is recommended. If negative there is no indication of a pregnancy termination. If below 10% or above 90% genetic counseling may be straightforward. Between these values it’s unsafe to be determinative of the future. Relatively to autosomal recessive trait an evaluation is required when previous diagnosis in offspring or positive family history, recurring to amniocentesis to test the fetus. If the fetus has mutated DNA in both alleles certainly will be affected, leaving the geneticist and the family the final decision.

## Treatment

Specific mitochondrial support: currently there is no sustained evidence of effective interventions in mitochondrial diseases [17], except few are “treatable” mitochondrial diseases [95]. In patients with *SLC19A3* mutations some improvement is seen, especially in early presentation, in thiamine (10-20 mg/kg/day) and biotin (10-15 mg/kg/day) oral supplementation [96]. They seem to enhance some transporters modulated by *SCL19A3* [50]. The same is observed in high doses supplementation of thiamine (30-40 mg/kg/day) in patients with *PDHA1* deficiency by stabilizing the PDHc [96, 97]. Primary thiamine defects, such as *TPK1* deficiency, are also under treatment option in thiamine reposition in 20 mg/kg/day [96]. CoQ10 is another that is reasonably effective in mitochondrial disorders, besides its primary deficiency, providing recovery of neurologic symptoms. Although acting in electron transfer from complexes in ETC and playing an antioxidant part in many cellular processes [98] and even higher dose are well tolerated even benefits are discussed. The recommended dose of CoQ10 is 10-30 mg/kg/day via oral supplementation [96].

General treatment: some general treatments have been studied along with specific therapies in order to ameliorate symptoms, avoid disease progression, clinical decompensation and enhance quality of life. A derivate from CoQ10, idebenone, was introduced due to easier tolerance profile and mitochondrial uptake, allowing results by a better improvement of ATP generation [99]. Recently, an alternative drug - EPI-743, a Coenzyme Q10 analog - has been developed and proved to have a better therapeutic profile. The first trial reported a promising improvement in patients with genetically confirmed mitochondrial disease, leading to a second trial. The patients who remained until the study was completed evolved with improvement and the one that discontinued medication fell back to symptoms [100]. EPI-743 increases intracellular glutathione levels by enhancing reduced intracellular glutathione stock [101]. Epilepsy is a common feature of LS demanding antiepileptic drugs to be considered to control the episodes. The choice of an antiepileptic drug must be taken considering the collateral profile and the benefits associated with the medication. It is already know that valproic acid, phenobarbital and phenytoin have deleterious effects in ETC, yet in case of uncontrolled prolonged crises (epileptic status) the option is based on risk - benefit of the emergency or medication availability [102]. Along with medications, some alternatives in dietary modification might be helpful. In some inhibitors of mitochondrial metabolism defects (*HIBCH, ECHS1*) a valine-restricted diet is an option, even with unclear responses so far [96]. Another option is the ketogenic diet is a dietary modality that avoids carbohydrates and focuses on lipids and proteins, producing ketone bodies instead of glucose as a cellular fuel [103]. Despite trials, ketogenic diet is still controversial to mitochondrial disease [95]. In *ETHE1* deficiency metronidazole associated to N-acetyl-cysteine might reduce sulfides and improve clinical and biochemical status [14]. As until now there is no curative treatment the available choices must be optimized in order to best benefit the patients in providing care and leaves an open door to new studies in finding new and, hopefully, definitive therapeutic option.

New therapies - as reproductive assistance and gene therapy are emerging and slowly expanding the resources field availability. Zhang J. et al described a live born baby resultant of an oocyte spindle transfer. The mother carried homoplasmy m.8993 T > G in germline cells, had multiple pregnancy losses, positive family history of LS, and decided to conceive again mitigating risks of a new unhealthy baby. Tests in donor material and in blastocyst for mutation load were made and came in safe range percentage. After birth, new samples were collected and preserved in safe levels [104]. This is an alternative to mothers who are known to harbor a pathogenic mitochondrial mutation. As far as direct intervention, gene therapy is being developed for LS. Allotopic gene expression was reported by Manfredi G. et al in mammalian nucleus to rescue the *MTATP6* function in producing ATP successfully achieved [105]. Tanaka M. et al reported the use of a restriction endonuclease to mitochondria managing to eliminate only in mutated mtDNA and preserving wild type in cultured cells [106]. New techniques like TALENS (Transcription activator-like effector nucleases) to mitochondria (mito-TALEN’s) were also reported to be quite selective in patient-derived cells harboring m.14459 G > A [107]. Di Meo I. et al seek to repair neurologic affected areas by LS in animal model and reported only partial improvement in neurologic symptoms due to a selective distribution of the vector to certain brain areas requiring further studies to have better results [108].

## Prognosis

Even with diagnosis and management improvements, the survival remains poor. As a heterogeneous disease, it takes time for diagnosis and little chances of having specific interventions as they are needed. Sofou K. et al presented a cohort of 130 patients with early presentation, described their natural history and identified possible factors of impact on survival as disease severity. In their cohort premature births, interventions in immediate neonatal period were present in little frequency yet pointing that prenatal presentation is still true. Pathological findings at neonatal period and epilepsy were described to be related to more frequent relapses as the age of onset, brainstem abnormalities in neuroimaging studies and hospitalization in Intensive Unit Care due to acute exacerbations were identified as poor prognosis predictors. Some groups published surviving rates that rarely encompass the third decade of life. The mortality is precocious not only in LS, but in mitochondrial diseases in general, due to complications and deterioration of organs and systems. Delayed diagnosis compromising optimal care also contributes to poor prognosis [109].

## Conclusion

LS is a neurodegenerative disease with broad phenotype and genotype presentations, in expanding picture. Besides diagnosis tools progresses, like NGS, new serum biomarkers and functional studies, treatment options are still restricted. It is still a challenge to make diagnosis and offer an optimal treatment due to its heterogeneous causes, therefore enhancing the need of new trials and further research to enhance quality of life and better providing care to the patients.

## Data Availability

Online data research does not involved human material itself. The imaging used has the copyrights and the identifier information accordingly to the website instructions.

## Abbreviations

*ADAR*: Adenosine deaminase RNA Specific
ADP: Adenosine Diphosphate
ATP synthase: Complex V; *MTATP6*: ATP synthase 6
ATP: Adenosine Triphosphate
*BBGD*: Biotine-responsive basal ganglia disease
BCKDH: Branched-chain α-ketoacid dehydrogenase complex
*BCS1L*: Ubiquinol-cytochrome c reductase complex chaperone
*BOLA3*: BOLA family member 3
*BTD*: Biotinidase
*C12Orf65*: Chromosome 12 open reading frame 65
*C17ORF89*: NADH:ubiquinone oxidoreductase complex I, assembly factor 8 (NDUFAF8)
*CLPB*: Caseinolytic peptidase B protein homolog
CNS: Central nervous system
Complex I: NADH (Nicotinamide Adenine Dinucleotide Dehydrogenase)/ubiquinone oxireductase
Complex III: Ubiquinol:cytochrome c oxidoreductase
CoQ10: Coenzyme Q10
*COQ9*: Coenzyme Q9 homolog
*COX*: Cytocrome c oxydase (complex IV)
*COX10*: Cytochrome c oxidase assembly homolog COX10
*COX15*: Cytochrome c oxidase assembly homolog COX15
*COX8A*: Cytochrome c oxidase subunit 8a
CSF: Cerebral spinal fluid
CVB: Chorionic villus biopsy
*DLAT*: Dihydrolipoamide S-acetyltransferase
*DLD*: Dihydrolipoamide dehydrogenase
DNA: Deoxyribonucleic acid
*DNM1L*: Dynamin 1 like protein
*EARS2*: Glutamyl-tRNA synthetase 2
EPI-743: *Vatiquinone*
ETC: Electrons Transport Chain
*ETHE1*: Persulfide dioxygenase
FADH/FADH_2_: Reduced forms of Flavin Adenine Dinucleotide (FAD)
*FARS2*: Phenylalanyl-tRNA synthetase
*FBLX4*: F-Box and leucine rich repeat protein 4
FGF-21: Fibroblast growth factor 21
*FOXRED1*: FAD-dependent oxidoreductase domain-containing protein 1
GDF-15: Growth and differentiation factor 15
*GFM1*: *Mitochondrial e*longation factor g 1
*GFM2*: Mitochondrial elongation factor g 2
GRACILE: Growth Retardation, Aminoaciduria, Cholestasis, Iron overload, Lactic acidosis, and Early death
*GTPBP3*: GTP binding protein 3
*HIBCH*: 3-hydroxyisobutyryl-CoA hydrolase
*IARS2*: Isoleucyl-tRNA synthetase 2
KGDH: α-ketoglutarate dehydrogenase
LHON: Leber’s Hereditary Optic Neuropathy
*LIAS*: Lipoic acid synthetase
*LIPT1*: Lipoyltransferase 1
*LIPT1*: Lipoyltransferase 1 deficiency
LLS: Leigh-like Syndrome
*LRPPRC*: Leucine rich pentatricopeptide repeat containing
LS: Leigh Syndrome
MEGDEL: 3-Methylglutaconic aciduria, Deafness, Encephalopathy and Leigh-like syndrome
MELAS: Mitochondrial Encephalomyopathy, Lactic Acidosis and Stroke-like episodes
MERRF: Myoclonic Epilepsy with Ragged Red Fibers
*MFN2*: Mitofusin-2
MILS: Maternally Inherited Leigh Syndrome
Mito-TALENS: mitochondrial transcription activator-like effector nucleases
*MPV17*: Mitochondrial inner membrane protein *17*
MRI: Magnetic resonance imaging
mRNA: Mitochondrial RNA
*MRPS34*: Mitochondrial ribosomal protein s34
*MRPS39*: Mitochondrial ribosomal protein s39
mtDNA: Mitochondrial DNA
*MTFMT*: Mitochondrial methionyl-tRNA formyltransferase
*MTND 1–6*: Mitochondrial encoded ND genes 1 to 6
*MTND1*: Mitochondrial encoded ND gene 1
*MTND3*: Mitochondrial encoded ND gene 3
*MTND5*: Mitochondrial encoded ND gene 5
*MTND6*: Mitochondrial encoded ND gene 6
*MTTK*: Mitochondrial tRNA for lysine
*MTTL1*: Mitochondrial tRNA for leucine
mt-tRNA: Mitochondrial transfer RNA
NADH: Reduced Nicotinamide Adenine Dinucleotide (NAD)
NARP: Neuropathy, Ataxia and Retinititis Pigmentosa
*NARS2*: Asparaginyl-tRNA synthetase 2
nDNA: Nuclear DNA
*NDUFA1*: NADH:ubiquinone oxidoreductase complex I alpha subcomplex subunit 1
*NDUFA10*: NADH:ubiquinone oxidoreductase complex I alpha subcomplex subunit 10
*NDUFA12*: NADH:ubiquinone oxidoreductase complex I alpha subcomplex subunit 12
*NDUFA2*: NADH:ubiquinone oxidoreductase complex I alpha subcomplex subunit 2
*NDUFA4*: NADH:ubiquinone oxidoreductase complex I alpha subcomplex subunit 4
*NDUFA4*: NADH-ubiquinone oxidoreductase 1 alpha subcomplex 4
*NDUFA9*: NADH:ubiquinone oxidoreductase complex I alpha subcomplex subunit 9
*NDUFAF2*: NADH:ubiquinone oxidoreductase complex I, assembly factor 2
*NDUFAF4*: NADH/ubiquinone oxidoreductase complex assembly factor 4
*NDUFAF4*: NADH:ubiquinone oxidoreductase complex I, assembly factor 4
*NDUFAF5*: NADH:ubiquinone oxidoreductase complex I, assembly factor 5
*NDUFAF6*: NADH:ubiquinone oxidoreductase complex I, assembly factor 6
*NDUFB8*: NADH:ubiquinone oxidoreductase 1 beta subcomplex subunit 8
*NDUFS1*: NADH:ubiquinone oxidoreductase Fe-S protein 1
*NDUFS2*: NADH:ubiquinone oxidoreductase Fe-S protein 2
*NDUFS3*: NADH/ubiquinone oxidoreductase core subunit s3
*NDUFS3*: NADH:ubiquinone oxidoreductase Fe-S protein 3
*NDUFS4*: NADH:ubiquinone oxidoreductase Fe-S protein 4
*NDUFS7*: NADH:ubiquinone oxidoreductase Fe-S protein 7
*NDUFS8*: NADH:ubiquinone oxidoreductase Fe-S protein 8
*NDUFV1*: NADH/ubiquinone oxidoreductase core subunit v1
*NDUFV1*: NADH:ubiquinone oxidoreductase flavoprotein 1
*NDUFV2*: NADH:ubiquinone oxidoreductase flavoprotein 2
NGS: Next Generation Sequencing
*NUBP*: Nucleotide-binding protein-like
*NUP62*: Nucleoporin p62
OXPHOS: Oxidative Phosphorylation
*PDHA1*: Pyruvate dehydrogenase alpha-1
PDHB: Pyruvate dehydrogenase e1 beta subunit
PDHC: Pyruvate desydrogenase complex
*PDHX*: Pyruvate dehydrogenase complex component x
*PDSS2*: Decaprenyl-diphosphate synthase subunit 2
PEO: Progressive External Ophthalmoplegia
*PET100*: Cytochrome c oxidase chaperone PET100
*PNPT1*: Polyribonucleotide nucleotidyltransferase 1
*POLG*: DNA polymerase gamma
*POLG2*: DNA polymerase gamma accessory subunit
PolyPhen-2: Polymorphism Phenotyping v2
*RANPB2*: RAN-binding protein 2
*RNASEH1*: Ribonuclease H1
RNA-seq: RNA sequencing
*RRM2B*: Ribonucleotide reductase regulatory tp53 inducible subunit m2b
rWES: Rapid Whole Exome Sequencing
rWGS: Rapid Whole Genome Sequencing
*SCL25A4*: Solute carrier family 25 member 4
*SCO2*: SCO Cytochrome c oxidase assembly protein 2
*SDH*: Succinate dehydrogenase (complex II)
*SDHA*: Succinate dehydrogenase complex subunit a
*SDHA1*: Succinate dehydrogenase flavoprotein subunit
*SDHB*: Succinate dehydrogenase complex subunit b
SIFT: *Scale-Invariant Feature Transform*
*SLC19A3*: Solute carrier family 19 member 3
*SLC25A4*: Solute carrier family 25 member 4
*SLC39A8*: Solute carrier family 39 member 8
*SPG7*: Matrix ATPases associated with diverse cellular activities (AAA) peptidase subunit, paraplegin
*SUCLA2*: Succinate-CoA ligase, ADP-forming, beta subunit
*SUCLG1*: Succinate-CoA ligase, alpha subunit
*SURF1*: Surfeit locus protein 1
*TACO1*: Translational activator of cytochrome c oxidase I
TALENS: *T*ranscription activator-like effector nucleases
TCA: Tricarboxylic Acid cycle
*TPK1*: Thiamin pyrophosphokinase 1
*TPP*: Thiamine pyrophosphate
*TRMU*: tRNA 5-methylaminomethyl-2-thiouridylate methyltransferase
*TSFM*: Translation elongation factor
*TTC19*: Tetratricopeptide repeat domain/protein 19
*TWNK*: Twinkle mitochondrial DNA helicase
*UQCRQ*: Ubiquinol-cytochrome c reductase complex III subunit VII
WES: Whole Exome Sequencing

## References

[1] Crimi M, O’Hearn SF, Wallace DC, Comi GP (2005). Molecular research technologies in mitochondrial diseases: the microarray approach. IUBMB Life.

[2] Zhang H, Burr SP, Chinnery PF (2018). The mitochondrial DNA genetic bottleneck: inheritance and beyond. Essays Biochem.

[3] DiMauro S, Schon EA (2003). Mitochondrial respiratory-chain diseases. N Engl J Med.

[4] Hawswirth WW, Laipis PJ (1982). Mitochondrial DNA polymorphism in a maternal lineage of Holstein cows. Proc.Natl.Acad.Sci..

[5] Chinnery PF, Thorburn DR, Samuels DC, White SL, Dahl HM, Turnbull DM (2000). The inheritance of mitochondrial DNA heteroplasmy: random drift, selection or both. Trends Genet.

[6] Rebolledo-Jaramillo B, Su MS-W, Stoler N, McElhoe JA, Dickins B, Blankenberg D (2014). Maternal age effect and severe germ-line bottleneck in the inheritance of human mitochondrial DNA. Proc Natl Acad Sci.

[7] Alston CL, Rocha MC, Lax NZ, Turnbull DM, Taylor RW (2017). The genetics and pathology of mitochondrial disease. J Pathol.

[8] El-Hattab AW, Scaglia F (2013). Mitochondrial DNA depletion syndromes: review and updates of genetic basis, manifestations, and therapeutic options. Neurotherapeutics..

[9] Carrol CJ, Brilhante V, Suomalainen A (2014). Next-generation sequencing for mitochondrial disorders. Br J Pharmacol.

[10] Leigh D (1951). Subacute necrotizing Encephalomyelopathy in an infant. J Neurol Neurosurg Psychiatry.

[11] Baertling F, Rodenburg RJ, Schaper J, Smeitink JA, Koopman WJ, Mayatepek E (2014). A guide to diagnosis and treatment of Leigh syndrome. J Neurol Neurosurg Psychiatry.

[12] Fassone E, Rahman S (2012). Complex I deficiency: clinical features, biochemistry and molecular genetics. J Med Genet.

[13] Rahman J, Noronha A, Thiele I, Rahman S (2017). Leigh map: a novel computational diagnostic resource for mitochondrial disease. Ann Neurol.

[14] Lake NJ, Compton AG, Rahman S, Thorburn DR (2016). Leigh syndrome: one disorder, more than 75 monogenic causes. Ann Neurol.

[15] Baertling F, Klee D, Haak TB, Prokisch H, Meitinger T, Mayatepek E (2016). The many faces of paediatric mitochondrial disease on neuroimaging. Childs Nerv Syst.

[16] Mckelvie P, Infeld B, Marotta R, Chin J, Thorburn D, Collins S (2012). Late-adult onset Leigh syndrome. J Clin Neurosci.

[17] Gerards M, Sallevelt SC, Smeets HJ (2016). Leigh syndrome: resolving the clinical and genetic heterogeneity paves the way for treatment options. Mol Genet Metab.

[18] Meyer JN, Hartman JH, Mello DF (2018). Mitochondrial toxicity. Toxicol Sci.

[19] Bricout M, Grévent D, Lebre AS, Rio M, Desguerre I, De Lonlay P (2014). Brain imaging in mitochondrial respiratory chain deficiency: combination of brain MRI features as a useful tool for genotype/phenotype correlations. J Med Genet.

[20] Namavar Y, Barth PG, Kasher PR, van Ruissen F, Brockmann K, Bernet G (2011). Clinical, neuroradiological and genetic findings in pontocerebellar hypoplasia. Brain..

[21] Lin J, Faria EC, Da Rocha AJ, Masrhua MR, LCP V, Scheper GC (2012). Leukoencephalopathy with brainstem and spinal cord and normal lactate: a new mutation in *DARS2* gene. J Neurol.

[22] Cheldi A, Ronchi D, Bordoni A, Bordo B, Lanfranconi S, Bellotti MG (2013). POLG1 mutations and stroke like episodes: a distinct clinical entity rather than an atypical MELAS syndrome. BMC Neurol.

[23] Valayannopoulos V, Haudry C, Serre V, Barth M, Boddaert N, Arnoux J-B (2010). New *SUCLG1* patients expanding the phenotypic spectrum of this rare cause of mild methylmalonic aciduria. Mitochondrion..

[24] Incecik F, Herguner OM, Besen S, Bozdogan ST, Mungan NO (2018). Late-onset Leigh syndrome due to NDUFV1 mutation in a 10-year-old boy initially presenting with ataxia. J Pediatr Neurosci.

[25] Baertling F, Sánchez-Caballero L, van den Brand MAM, Wintjes LT, Brink M, van den Brandt FA (2017). NDUFAF4 variants are associated with Leigh syndrome and cause a specific mitochondrial complex I assembly defect. Eur J Hum Genet.

[26] Lou X, Shi H, Wen S, Li Y, Wei X, Xie J, Ma L (2018). A novel NDUFS3 mutation in a chinese patient with severe Leigh syndrome. J Hum Genet.

[27] Spangenberg L, Graña M, Greif G, Suarez-Rivero JM, Krysztal K, Tapie A (2016). 3697G>A in MT-ND1 is a causative mutation in mitochondrial disease. Mitochondrion..

[28] Ma YY, Wu TF, Liu YP, Wang Q, Song JQ, Li XY (2013). Genetic and biochemical findings in chinese children with Leigh syndrome. J Clin Neurosci.

[29] Ruiter EM, Siers MH, van den Elzen C, van Engelen BG, JAM S, Rodenburg RJ (2007). The mitochondrial 13513 A>G mutation is most frequent in Leigh syndrome combined with reduced complex I activity, optic atrophy and/or Wolff-Parkinson White. Eur J Hum Genet.

[30] Sudo A, Honzawa S, Nonaka I, Goto Y (2004). Leigh syndrome caused by mitochondrial DNA G13513A mutation: frequency and clinical features in Japan. J Hum Genet.

[31] Dermaut B, Seneca S, Dom L, Smets K, Ceulemans L, Smet J (2010). Progressive myoclonic epilepsy as an adult-onset manifestation of Leigh syndrome due to m.14487T>C. J Neurol Neurosurg Psychiatry.

[32] Courage C, Jackson CB, Hahn D, Euro L, Nuoffer JM, Gallati S (2017). SDHA mutation with dominant transmission results in complex II deficiency with ocular, cardiac, and neurologic involvement. Am J Med Genet A.

[33] Fernández-Vizarra E, Zeviani M (2015). Nuclear gene mutations as the cause of mitochondrial complex III deficiency. Front Genet.

[34] Baker RA, Priestley JRC, Wilstermann AM, Reese KJ, Mark PR (2019). Clinical spectrum of BCS1L mitopathies and their underlying structural relationships. Am J Med Genet A.

[35] Nogueira C, Barros J, Sa MJ, Azevedo L, Taipa R, Torraco A (2013). Novel TTC19 mutation in a family with severe psychiatric manifestations and complex III deficiency. Neurogenetics..

[36] Atwal PS (2014). Mutations in the complex III assembly factor tetratricopeptide 19 gene TTC19 are a rare cause of Leigh syndrome. JIMD Rep.

[37] Rahman S, Clarke CF, Hirano M (2012). 176th ENMC international workshop: diagnosis and treatment of coenzyme Q10 deficiency. Neuromuscul Disord.

[38] Van Maldergem L, Trijbels F, DiMauro S, Sindelar PJ, Musumeci O, Janssen A (2002). Coenzyme Q–responsive Leigh’s encephalopathy in two sisters. Ann Neurol.

[39] Glover EI, Martin J, Maher A, Thornhill RE, Moran GR, Tarnopolsky MA (2010). A randomized trial of coenzyme Q10 in mitochondrial disorders. Muscle Nerve.

[40] Baertling F, Mayatepek E, Distelmaier F (2013). Hypertrichosis in presymptomatic mitochondrial disease. J Inherit Metab Dis.

[41] Debray FG, Morin C, Janvier A, Villeneuve J, Maranda B, Laframboise R (2011). LRPPRC mutations cause a phenotypically distinct form of Leigh syndrome with cytochrome c oxidase deficiency. J Med Genet.

[42] Cui J, Wang L, Ren X, Zhang Y, Zhang H (2019). LRPPRC: A multifunctional protein involved in energy metabolism and human disease. Front Physiol.

[43] Pitceathly RD, Rahman S, Wedatilake Y, Polke JM, Cirak S, Foley AR (2013). NDUFA4 mutations underlie dysfunction of a cytochrome *c* oxidase subunit linked to human neurological disease. Cell Rep.

[44] Ganetzky RD, Stendel C, McCormick EM, Zolkipli-Cunningham Z, Goldstein AC, Klopstock T (2019). MT-ATP6 mitochondrial disease variants: phenotypic and biochemical features analysis in 218 published cases and cohort of 14 new cases. Hum Mutat.

[45] Vilarinho L, Leão E, Barbot C, Santos M, Rocha H, Santorelli FM (2000). Clinical and molecular studies in three portuguese mtDNA T8993G families. Pediatr Neurol.

[46] Lopes T, Coelho M, Bordalo D, Bandeira A, Bandeira A, Vilarinho L (2018). Sindrome de Leigh: a propósito de um caso clínico com mutação no DNA mitocondrial. Rev Paul Pediatr.

[47] Wei Y, Cui L, Peng B (2018). Mitochondrial DNA mutations in late-onset Leigh syndrome. J Neurol.

[48] Carrozzo R, Tessa A, Vázquez-Memije ME, Piemonte F, Patrono C, Malandrini A (2001). The T9176G mtDNA mutation severely affects ATP production and results in Leigh syndrome. Neurology..

[49] Nogueira C, Carrozo R, Vilarinho L, Santorelli FM (2011). Infantile-onset disorders of mitochondrial replication and protein synthesis. J Child Neurol.

[50] Goldstein A, Falk MJ. Mitochondrial DNA deletion syndromes. GeneReviews®. Seattle: University of Washington, Seattle. p. 1993–2019. Available from: https://www.ncbi.nlm.nih.gov/books/NBK1203/. Acessed 19 Sept 2019. 20301382

[51] Miller C, Wang L, Ostergaard E, Dan P, Saada A (2011). The interplay between SUCLA2, SUCLG2, and mitochondrial DNA depletion. Biochim Biophys Acta.

[52] Sequeira S, Rodrigues M, Jacinto S, Wevers RA, Wortmann SB (2017). MEGDEL syndrome: expanding the phenotype and new mutations. Neuropediatrics..

[53] Wortmann SB, Vaz FM, Gardeitchik T, Vissers LE, Renkema GH, Schuurs-Hoeijmakers JH (2012). Mutations in the phospholipid remodeling gene SERAC1 impair mitochondrial function and intracellular cholesterol trafficking and cause dystonia and deafness. Nat Genet.

[54] Taanman JW, Rahman S, Pagnamenta AT, Morris AA, Bitner-Glindzicz M, Wolf NI (2009). Analysis of mutant DNA polymerase gamma in patients with mitochondrial DNA depletion. Hum Mutat.

[55] Kinghorn KJ, Kaliakatsos M, Blakely EL, Taylor RW, Rich P, Clarke A (2013). Hypertrophic olivary degeneration on magnetic resonance imaging in mitochondrial syndromes associated with POLG and SURF1 mutations. J Neurol.

[56] Souza PVS, Bortholin T, Teixeira CAC, Seneor DD, Marin VDGB, Dias RB (2019). Leigh syndrome caused by mitochondrial DNA-maintenance defects revealed by whole exome sequencing. Mitochondrion..

[57] Distelmaier F, Huppke P, Pieperhoff P, Amunts K, Schaper J, Morava E (2014). Biotin-responsive basal ganglia disease: a treatable differential diagnosis of Leigh syndrome. JIMD Rep.

[58] Gerards M, Kamps R, van Oevelen J, Boesten I, Jongen E, de Koning B (2013). Exome sequencing reveals a novel Moroccan founder mutation in SLC19A3 as a new cause of early-childhood fatal Leigh syndrome. Brain..

[59] Haack TB, Klee D, Strom TM, Mayatepek E, Meitinger T, Prokisch H (2014). Infantile Leigh-like syndrome caused by SLC19A3 mutations is a treatable disease. Brain..

[60] Alfadhel M (2017). Early infantile Leigh-like SLC19A3 gene defects have a poor prognosis: report and review. J Cent Nerv Syst Dis.

[61] Ortigoza-Escobar JD, Molero-Luis M, Arias A, Oyarzabal A, Darin N, Serrano M (2016). Free-thiamine is a potential biomarker of thiamine transporter-2: a treatable cause of Leigh syndrome. Brain..

[62] Schiff M, Miné M, Brivet M, Marsac C, Elmaleh-Bergés M, Evrard P (2006). Leigh’s disease due to a new mutation in the PDHX gene. Ann Neurol.

[63] van Dongen S, Brown RM, Brown GK, Thorburn DR, Boneh A (2015). Thiamine-responsive and non-responsive patients with PDHC-E1 deficiency: a retrospective assessment. JIMD Rep.

[64] Soreze Y, Boutron A, Habarou F, Barnerias C, Nonnenmacher L, Delpech H (2013). Mutations in human lipoyltransferase gene LIPT1 cause a Leigh disease with secondary deficiency for pyruvate and alpha-ketoglutarate dehydrogenase. Orphanet J Rare Dis.

[65] Stowe RC, Sun Q, Elsea SH, Scaglia F (2018). LIPT1 deficiency presenting as early infantile epileptic encephalopathy, Leigh disease, and secondary pyruvate dehydrogenase complex deficiency. Am J Med Genet A.

[66] Rabier D, Diry C, Rotig A, Rustin P, Heron B, Bardet J (1998). Persistent hypocitrullinaemia as a marker for mtDNA NARP T 8993 G mutation?. J Inherit Metab Dis.

[67] Benavente-Talavera SA, Cabanillas-Burgos LY, Vera-Portilla AF (2018). Síndrome de NARP en paciente pediátrico: reporte de un caso. Revista Médica Basadrina.

[68] Bugiardini E, Pope S, Feichtinger RG, Poole OV, Pittman AM, Woodward CE (2019). Utility of whole blood thiamine pyrophosphate evaluation in *TPK1*-related diseases. J Clin Med.

[69] Lin DD, Crawford TO, Barker PB (2003). Proton MR spectroscopy in the diagnostic evaluation of suspected mitochondrial disease. Am J Neuroradiol.

[70] Sijens PE, Smit GP, Rödiger LA, van Spronsen FJ, Oudkerk M, Rodenburg RJ (2008). MR spectroscopy of the brain in Leigh syndrome. Brain Dev.

[71] Lebre AS, Rio M, Faivre d’Arcier L, Vernerey D, Landrieu P, Slama A (2011). A common pattern of brain MRI imaging in mitochondrial diseases with complex I deficiency. J Med Genet.

[72] Montero R, Yubero D, Villarroya J, Henares D, Jou C, Rodríguez MA (2016). GDF-15 is elevated in children with mitochondrial diseases and is induced by mitochondrial disfunction. PLoS One.

[73] Yatsuga S, Fujita Y, Ishii A, Fukumoto Y, Arahata Y, Kakuma T (2015). Growth differentiation factor 15 as a useful biomarker for mitochondrial disorders. Ann Neurol.

[74] Lehtonen JM, Forsström S, Bottani E, Viscomi C, Baris OR, Isoniemi H (2016). FGF21 is a biomarker for mitochondrial translation and mtDNA maintenance disorders. Neurology..

[75] Morovat A, Weerasinghe G, Nesbitt V, Hofer M, Agnew T, Quaghebeur G (2017). Use of FGF-21 as a biomarker of mitochondrial disease in clinical practice. J Clin Med.

[76] Tsygankova PG, Itkis YS, Krylova TD, Kurkina MV, Bychkov IO, Ilyushkina AA (2019). Plasma FGF-21 and GDF-15 are elevated in different inherited metabolic diseases and are not diagnostic for mitochondrial disorders. J Inherit Metab Dis.

[77] Janssen AJ, Trijbels FJ, Sengers RC, Wintjes LT, Ruitenbeek W, Smeitink JA (2006). Measurement of the energy-generating capacity of human muscle mitochondria: diagnostic procedure and application to human pathology. Clin Chem.

[78] Wortmann SB, Mayr JA, Nuoffer JM, Prokisch H, Sperl W (2017). A guideline for the diagnosis of pediatric mitochondrial disease: the value of muscle and skin biopsies in the genetics era. Neuropediatrics..

[79] Adzhubei IA, Schmidt S, Peshkin L, Ramensky VE, Gerasimova A, Bork P (2010). A method and server for predicting damaging missense mutations. Nat Methods.

[80] Kumar P, Henikoff S, Ng PC (2009). Predicting the effects of coding non-synonymous variants on protein function using the SIFT algorithm. Nat Protoc.

[81] Schwarz JM, Rödelsperger C, Schuelke M, Seelow D (2010). MutationTaster evaluates disease-causing potential of sequence alterations. Nat Methods.

[82] Thorburn DR, Rahman J, Rahman S. Mitochondrial DNA-associated Leigh syndrome and NARP. GeneReviews®. Seattle: University of Washington, Seattle. p. 1993–2019. Available from: https://www.ncbi.nlm.nih.gov/books/NBK1173/. Acessed 19 Sept 2019. 20301352

[83] Kremer LS, Wortmann SB, Prokisch H (2018). “Transcriptomics”: molecular diagnosis of inborn errors of metabolism via RNA-sequencing. J Inherit Metab Dis.

[84] Nogueira C, Silva L, Pereira C, Vieira L, Teles EL, Rodrigues E (2019). Targeted next generation sequencing identifies novel pathogenic variants and provides molecular diagnosis in a cohort os pediatric and adult patients with unexplained mitochondrial dysfunction. Mitochondrion..

[85] Pronicka E, Piekutowska-Abramczuk D, Ciara E, Trubicka J, Rokicki D, Karkucińska-Więckowska A (2016). New perspective in diagnostics of mitochondrial disorders: two years’experience with whole-exome sequencing at national paediatric centre. J Transl Med.

[86] Taylor RW, Pyle A, Griffin H, Blakely EL, Duff J, He L (2014). Use of whole-exome sequencing to determine the genetic basis of multiple mitochondrial respiratory chain complexes deficiencies. JAMA..

[87] Saunders CJ, Miller NA, Soden SE, Dinwiddie DL, Noll A, Alnadi NA (2012). Rapid whole-genome sequencing for genetic disease diagnosis in neonatal intensive care units. Sci Transl Med.

[88] Farnaes L, Hildreth A, Sweeney NM, Clark MM, Chowdhury S, Nahas S (2018). Rapid whole-genome sequencing decreases infant morbidity and cost of hospitalization. NPJ Genom Med.

[89] Rohanizadegan M, Abdo SM, O’Donnel-Luria A, Mihalek I, Chen P, Sanders M (2017). Utility of rapid whole-exome sequencing in the diagnosis of Niemann-pick disease type C presenting with fetal hydrops and acute liver failure. Cold Spring Harb Mol Case Stud.

[90] Stenton SL, Kremer LS, Kopajtich R, Ludwig C, Prokisch H. The diagnosis of inborn errors of metabolism by an integrative “multiomics” approach: A perspective encompassing genomics, transcriptomics, and proteomics. J Inherit Metab Dis. 2019. p. 1–11. 10.1002/jimd.%2012130. 10.1002/jimd.12130 31119744

[91] White SL, Collins VR, Wolfe R, Cleary MA, Shanske S, DiMauro S (1999). Genetic counseling and prenatal diagnosis for the mitochondrial DNA mutations at nucleotide 8993. Am J Hum Genet.

[92] Nesbitt V, Alston CL, Blakely EL, Fratter C, Feeney CL, Poulton J (2014). A national perspective on prenatal testing for mitochondrial disease. Eur J Hum Genet.

[93] Craven L, Alston CL, Taylor RW, Turnbull DM (2017). Recent advances in mitochondrial disease. Annu Rev Genomics Hum Genet.

[94] Jacobs LJ, de Coo IF, Nijland JG, Galjaard RJ, Los FJ, Schoonderwoerd K (2005). Transmission and prenatal diagnosis of the T9176C mitochondrial DNA mutation. Mol Hum Reprod.

[95] Rahman S (2015). Emerging aspects of treatment in mitochondrial disorders. J Inherit Metab Dis.

[96] Distelmaier F, Haak TB, Wortmann SB, Mayr JA, Prikisch H (2017). Treatable mitochondrial diseases:cofactor metabolism and beyond. Brain..

[97] Brown G (2014). Defects of thiamine transport and metabolism. J Inherit Metab Dis.

[98] Hargreaves IP (2014). Coenzyme Q10 as a therapy for mitochondrial disease. Int J Biochem Cell Biol.

[99] Haginoya K, Kaneta T, Togashi N, Hino-Fukuyo N, Kobayashi T, Uematsu M (2016). FDG-PET study of patients with Leigh syndrome. J Neurol Sci.

[100] Martinelli D, Catteruccia M, Piemonte F, Pastore A, Tozzi G, Dionisi-Vici C (2012). EPI-743 reverses the progression of the pediatric mitochondrial disease--genetically defined Leigh syndrome. Mol Genet Metab.

[101] Murayama K, Shimura M, Liu Z, Okazaki Y, Ohtake A (2019). Recent topics: the diagnosis, molecular genesis, and treatment of mitochondrial diseases. J Hum Genet.

[102] Finsterer J, Mahjoub SZ (2011). Mitochondrial toxicity of antiepileptic drugs and their tolerability in mitochondrial disorders. Expert Opin Drug Metab Toxicol.

[103] Sampaio LP (2016). Ketogenic diet for epilepsy treatment. Arq Neuropsiquiatr.

[104] Zhang J, Liu H, Luo S, Lu Z, Chávez-Badiola A, Liu Z (2017). Live birth derived from oocyte spindle transfer to prevent mitochondrial disease. Reprod BioMed Online.

[105] Manfredi G, Fu J, Ojaimi J, Sadlock JE, Kwong JQ, Guy J (2002). Rescue of a deficiency in ATP synthesis by transfer of MTATP6, a mitochondrial DNA-encoded gene, to the nucleus. Nat Genet.

[106] Tanaka M, Borgeld HJ, Zhang J, Muramatsu S, Gong JS, Yoneda M (2002). Gene therapy for mitochondrial disease by delivering restriction endonuclease smaIl into mitochondria. J Biomed Sci.

[107] Bacman SR, Williams SL, Pinto M, Peralta S, Moraes CT (2013). Specific elimination of mutant mitochondrial genomes in patient-derived cells by mitoTALENs. Nat Med.

[108] Di Meo I, Marchet S, Lamperti C, Zeviani M, Viscomi C (2017). AAV9-based gene therapy partially ameliorates the clinical phenotype of a mouse model of Leigh syndrome. Gene Ther.

[109] Sofou K, De Coo IFM, Isohanni P, Ostergaard E, Naess K, De Meirleir L (2014). A multicenter study on Leigh syndrome: disease course and predictors of survival. Orphanet J Rare Dis.

