# Correction to: Molecular basis of Leigh syndrome: a current look

**DOI:** 10.1186/s13023-020-1351-7

**Published:** 2020-03-25

**Authors:** Manuela Schubert Baldo, Laura Vilarinho

**Affiliations:** Newborn screening, metabolism and genetics unit - human genetics department, Instituto Nacional de Saúde Doutor Ricardo Jorge (INSA), Porto, Portugal

**Correction to: Orphanet J Rare Dis (2020) 15:31**

**https://doi.org/10.1186/s13023-020-1297-9**

The original version of this article [1] unfortunately included an error to an author’s name. Author Manuela Schubert Baldo was erroneously presented as Manuela Baldo Schubert.

The author name has been updated in the original article and included in the author list of this Correction article.
